# Ozone Treatment Improves the Texture of Strawberry Fruit during Storage

**DOI:** 10.3390/antiox11050821

**Published:** 2022-04-22

**Authors:** Tomasz Piechowiak, Dagmara Migut, Radosław Józefczyk, Maciej Balawejder

**Affiliations:** 1 Department of Chemistry and Food Toxicology, Institute of Food Technology and Nutrition, University of Rzeszow, St. Cwiklinskiej 1a, 35-601 Rzeszow, Poland; rjozefczyk@ur.edu.pl (R.J.); mbalawejder@ur.edu.pl (M.B.); 2 Department of Crop Production, Institute of Agricultural Sciences, Land Management and Environmental Protection, University of Rzeszow, St. Zelwerowicza 4, 35-601 Rzeszow, Poland; dmigut@ur.edu.pl

**Keywords:** mitochondria, ozonation, strawberries, texture

## Abstract

The major aim of this study was to check whether a cyclic ozonation process will affect the preservation of the texture of strawberries stored at room temperature. Strawberry fruit was stored for 3 days at room temperature and ozonated with gaseous ozone at a concentration of 10 and 100 ppm for 30 min, every 12 h of storage. Research showed that the ozonation process inhibited the texture deterioration of the fruit during storage. The positive effect of ozone was directly related to the inhibition of the activity of enzymes involved in the degradation of the fruit cell walls, as well as indirectly from the improved energy metabolism of the fruit. The higher level of energy charge corresponded to the higher resistance of ozonated fruit to abiotic stress, leading to the maintenance of the integrity of cell membranes and, consequently, to maintaining good hardness of the fruit throughout the storage period.

## 1. Introduction

Over the past few years, changes in the lifestyle of the population, as well as the greater awareness of the importance of healthy foods in the diet, have led to the development of the market for immediately ready-to-eat food, especially minimally processed fruit and vegetables, free from synthetic additives [1]. This group includes strawberries (*Fragaria* × *ananassa* Duch.), which are a rich source of a wide range of bioactive compounds, such as vitamins, flavonoids, phenolic acids and minerals [2]. All of these compounds exert synergistic effects on human health and have a putative role in the prevention of many chronic and degenerative diseases associated with oxidative damage, such as cancer and heart disease [3].

Dynamic changes in the chemical composition and structure of the tissue of fruit during maturation, aging and processing are related to the content of polyphenols and vitamin C as well as to characteristics such as size, firmness, colour, taste and smell [4]. Although strawberries are characterised by a high metabolic rate, the main cause of fruit loss is the development of post-harvest diseases due to latent infections that occur in the field during the growing season and infections due to harvest and handling damage, which contribute to severe economic losses [5].

The largely decisive factor for the course of the storage process is the endemic microflora present on the plant material. Changes in this microflora can potentially affect its course. However, there are few factors that affect the microflora but do not deteriorate the quality of the plant material. In recent years, there has been an increase in the amount of research aimed at the use of safe and effective methods allowing the extension of storage time, while limiting the negative changes taking place within the plant material [1]. Some alternative antimicrobials include the action of heat, UV-C radiation, pulsing light, chlorine dioxide, ultrasound, natural antimicrobials and the use of edible coatings [6,7,8]. Ozonation can also be a technique used for this purpose [9]. Ozone is a substance with a strong bactericidal and fungicidal effect, which is widely used in the food industry to extend the shelf life of plant materials, including strawberry fruit [10,11,12,13,14,15]. Many studies conducted so far have shown that the use of ozone in the storage of plant materials reduces the growth of microorganisms that cause fruit spoilage and rotting, and it oxidises the ethylene released. Moreover, it has been proven that “light” ozonation conditions contribute to the inhibition of the depletion of biologically active substances or even an increase in their accumulation by elicitation [9,16,17].

Strawberries belong to the group of perishable fruit that are very susceptible to mechanical damage occurring during harvest and at various stages of technological processes or storage; therefore, texture is one of the most important quality attributes in their consumer assessment [18]. Strawberries are characterised by high heterogeneity and anisotropy of mechanical properties, which vary depending on the conditions and time of storage. The study of mechanical properties allows the determination of the quality of the plant material, its firmness and hardness, which is used to assess the quality of fruit after harvest, to predict the internal mechanical reaction (e.g., damage evolution) during various technological processes and storage, and to develop new solutions used in the technological process [19]. The research to date on the mechanics of the texture of strawberries can be divided into two aspects: that related to the measurements taken immediately after harvest and its impact on fruit damage, and studies monitoring the change in texture during fruit storage. Early identification of factors influencing plant material damage enables timely decisions on the production flow in order to significantly reduce economic losses [20].

In an aqueous environment, such as fresh plant materials, ozone breaks down into free radicals, mainly reactive oxygen species, an excess of which is toxic to plant cells [9,21]. The studies conducted so far show that one of the reasons for the softening of fruit in storage is both changes in the components of the cell walls in plant materials, as well as the occurrence of oxidative stress [22]. However, despite the increasing number of studies on the use of ozone in extending the shelf life of berries, there are no research data that fully explain the mechanism of action of gaseous ozone in relation to the texture of the fruit [13,14,15,23,24].

The major aim of this study was to check whether a cyclic ozonation process will affect the preservation of the texture of strawberries stored at room temperature. The scope of research work included: determining the texture of fruit ozonated with gas at a concentration of 10 and 100 ppm, examining changes in the activity of enzymes involved in fruit softening, and explaining the role of energy metabolism in shaping the texture of ozonated fruit.

## 2. Materials and Methods

### 2.1. Ozonation Process and Storage Experiment

The research material consisted of strawberry fruit (*Fragaria × ananassa* Duch.), Elkat variety, purchased directly from the producer on the day of harvest (June 2021). The fruit was fully coloured, with no signs of deterioration or mechanical damage. Before the ozonation process, strawberries (6 kg) of a uniform size were divided into three equal parts. The first part of the fruit was a control sample, while the remaining fruit was to be subjected to ozonation.

In this study, the same technology of cyclic ozonation and storage procedure were used as in the work of Piechowiak et al. [25] Briefly, the fruit was spread evenly on a perforated pallet which was then placed in a plastic container with a lid measuring 0.65 × 0.35 × 0.15 m (L × W × H). The fruit was ozonated with a gas concentration of 10 ppm (dose 1) and 100 ppm (dose 2) for 30 min, every 12 h of storage using a Korona L5 ozone generator (Piotrków Trybunalski, Poland) and the UV-106 M ozone analyser (2B Technologies, Boulder, CO, USA). At the same time, a control sample was set up, which consisted of fruit not subjected to the ozonation process. Both the ozonated samples and the control sample were stored at room temperature (20–22 °C, 65–70% of relative humidity) for 3 days. Samples for analysis were taken after 1, 2 and 3 days of storage, and then subjected to texture measurement and biochemical analyses. The experiment was performed in three replications.

### 2.2. Texture Analysis

The texture profile analysis (TPA) of strawberries was performed using Mecmesin MultiTest 2.5-i texture analyser (Slinfold, West Sussex, UK) following the appropriate methods on food products (compression test). The TPA criteria included hardness, cohesiveness, springiness, adhesion and gumminess [26]. The analysis was performed on six berries from each replicate of the experiment. The results were presented as mean value ± standard error (SE).

### 2.3. Determination of the Activity of Enzymes Related to Fruit Softening

The activity of β-galactosidase (β-Gal) and β-hexosaminidase (β-Hex) in fruit was determined by measuring the released *p*-nitrophenol from the appropriate substrate for β-Gal (4-nitrophenyl β-D-galactopyranoside) and β-Hex (4-nitrophenyl N-acetyl-beta-D-glucosaminide) [27,28]. One unit of β-Gal and β-Hex was the amount of enzyme causing an 0.01 increase in the absorbance at 400 nm for 1 min [U]. The activity of polygalacturonase (PG) was assayed by measuring the amount of the galacturonic acid produced as a results of pectin hydrolysis catalysed by PG [28]. One unit of PG activity was the amount of enzyme which catalyses the formation of 1 mg of galacturonic acid within 1 min [U].

### 2.4. Mitochondria Energy Metabolism Analysis

#### 2.4.1. Energy Charge Analysis

The levels of adenosine triphosphate (ATP) adenosine diphosphate (ADP) and adenosine monophosphate (AMP) were extracted from fruit tissue with perchloric acid according to the protocol presented by Zhou et al. [29] ATP, ADP and AMP concentration was determined spectophotometrically using the enzymatic method described by Tornheim and Schultz [30,31]. The contents of these compounds were expressed as mg per 1 kg of fresh mass of the fruit. The obtained results were used to determine the energy charge of the mitochondria, in accordance with the following equation:

EC = [ATP + 0.5 ADP]/[ATP + ADP + AMP].

#### 2.4.2. Oxidative Phosphorylation Enzymes Activity Assay

Mitochondria from strawberry fruit was isolated using differential centrifugation according to protocol described by Zhou et al. [29] The activities of the succinate dehydrogenase (SDH), cytochrome C oxidase (CCO) and H^+^-ATPase in mitochondria were analysed as described by Piechowiak et al. [32] One unit of SDH and CCO activity was defined as the amount of protein causing a 0.01 decrease in the absorbance, respectively, at 600 and 550 nm within 1 minute [U]. H^+^-ATPase activity was calculated as the amount of liberated phosphate after ATP hydrolysis within one minute [U].

#### 2.4.3. Reactive Oxygen Species Generation Analysis

The generation of superoxide radical anion by mitochondria (mt-O_2_^−^) was assayed using the spectrophotometric method which was based on the reduction in nitro blue tetrazolium by mt-O_2_^−^ leading to diformazan production [33]. The ability of mitochondria to hydrogen peroxide (mt-H_2_O_2_) production was analysed by means of 2′,7′-dichlorofluorescin diacetate as a fluorescent probe using the protocol presented by Piechowiak and Balawejder [33]. The results were expressed as an increase in the absorbance or fluorescence of the reaction mixture within 1 min, respectively, for mt-O_2_^−^ [ΔA min^−1^] and mt-H_2_O_2_ [ΔF min^−1^] production.

#### 2.4.4. Antioxidant Enzymes Activity

The activity of mitochondrial superoxide dismutase (mt-SOD) was analysed using the colorimetric method, which based on the measurement of the level of inhibition of epinephrine oxidation by mt-SOD. Additionally, 1 unit of mt-SOD activity is the amount of protein required to inhibit the initial rate of epinephrine oxidation by 50% within 1 min [U] [33]. The activity of mitochondrial glutathione peroxidase (mt-GPx) was measured according to the previously described and modified method [28]. One unit of enzyme activity was defined as the amount of the protein, causing the consumption of 1 µmol of glutathione (GSH) within 1 min [U].

### 2.5. Determination of Malondialdehyde Level in Fruit and Cell Membrane Electrolyte Leakage

The level of malondialdehyde (MDA) in the fruit was measured by a method based on the formation of MDA adduct with thiobarbituric acid (TBA) [34]. The MDA content was calculated based on the molar absorption coefficient for MDA (ε_532nm_ = 155 mM^−1^ cm^−1^).

The leakage of ions from the fruit samples was determined by measuring the conductivity of the solutions after immersing the fruit slices in distilled water and the slices heated in distilled water according to the protocol presented by Li-Qin et al. [35]

### 2.6. Statistical Analysis

The significance of differences between fruit samples was estimated using one-way ANOVA and Tukey’s test (α = 0.05, *n* = 9) in GraphPad.

## 3. Results

### 3.1. Changes in Fruit Texture during Storage at Room Temperature

**Figure 1:**
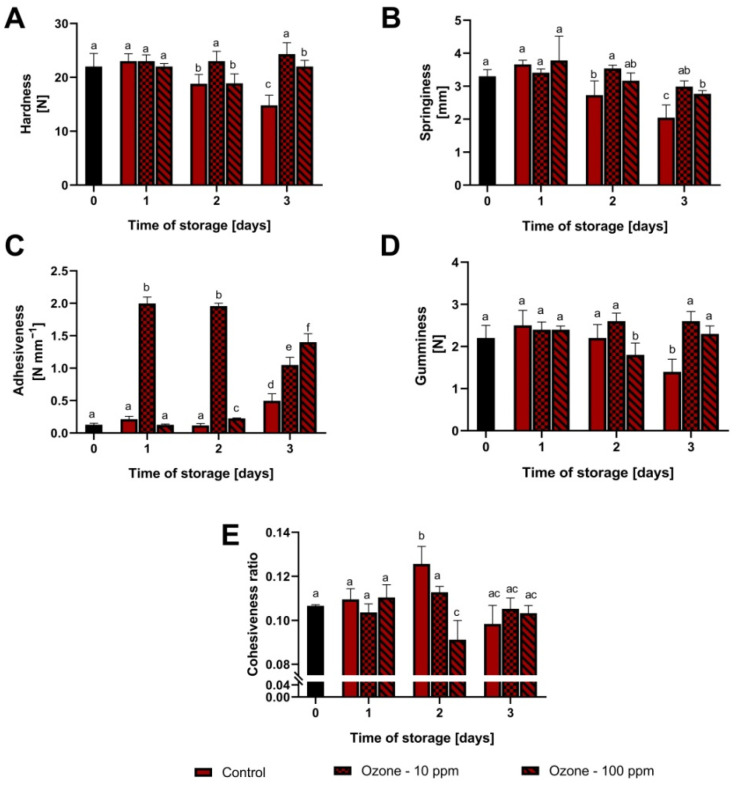
Effect of ozonation process on the texture profile of strawberry fruit during storage at room temperature. (**A**) fruit hardness; (**B**) fruit springiness; (**C**) fruit adhesiveness; (**D**) fruit gumminess; (**E**) fruit cohesiveness. Mean values with standard error (error bars), with the same lower case are not statistically significant according to the t-Tukey test (α = 0.05).

The relationship between the hardness (N) of the strawberry fruit and the applied ozone gas concentration during storage is presented in Figure 1A. On the first day of fruit storage, no significant differences were observed between those subjected to ozone concentrations and the control sample. With the passage of storage time, higher values of the analysed parameter were noted for ozonated fruit in relation to the control. On the second day of storage, significant differences were found between the control sample and the hardness of the fruit treated with ozone gas at a concentration of 10 ppm. In the case of the concentration of 100 ppm, no such relationship was found. On the third day of storage, the fruit with the concentration of 10 ppm showed no differences in relation to the first date of measurement. In the case of the concentration of 100 ppm, no significant difference was found in relation to the earlier date of measurement.

A similar relationship was observed in the case of springiness (mm) (Figure 1B). Over the duration of the experiment, the value of the analysed parameter for the control sample decreased, in contrast to the values obtained for the ozonated fruit. On the first day of storage, there were no significant differences between the values obtained for the ozonated fruit and the control sample, and on the second and third dates of measurement the fruit treated with ozone gas was characterised by higher elasticity values compared to the control, regardless of the ozone dose applied.

Figure 1C shows the relationship between the adhesiveness (N mm^−1^) of the tested fruit treated with ozone gas and its storage time. Fruit treated with a dose of 10 ppm of ozone gas was characterised by significantly higher values of the analysed parameter in relation to other variants of the experiment, on both the first and the second days of storage. On the third day of storage, an increase in the adhesiveness of the analysed samples was noted in each of the variants of the experiment, and the results obtained for the fruit that was treated with ozone gas were significantly higher compared to the control sample.

In the case of gumminess (N) (Figure 1D), on the first day of storage, no significant differences were found between the control sample and the fruit treated with ozonation. On the second day of storage, the highest value of the analysed parameter was observed for fruit with ozone concentration 10 ppm, but this value did not differ significantly from the control. On the third day, the fruit on which the ozonation process was carried out was characterised by significantly higher values of the analysed parameter as compared to the control, regardless of the ozone dose applied.

Figure 1E shows the relationship between cohesiveness and the storage time of fruit exposed to ozone gas in various doses. On the first date of measurement, there was no effect of using ozone gas on the value of the analysed parameter. On the second date, the control sample had the highest value, and on the third date, there was no relationship between the use of ozone gas and cohesiveness.

### 3.2. Changes in the Activity of β-Gal, β-Hex and PG in Strawberries during Storage

**Figure 2:**
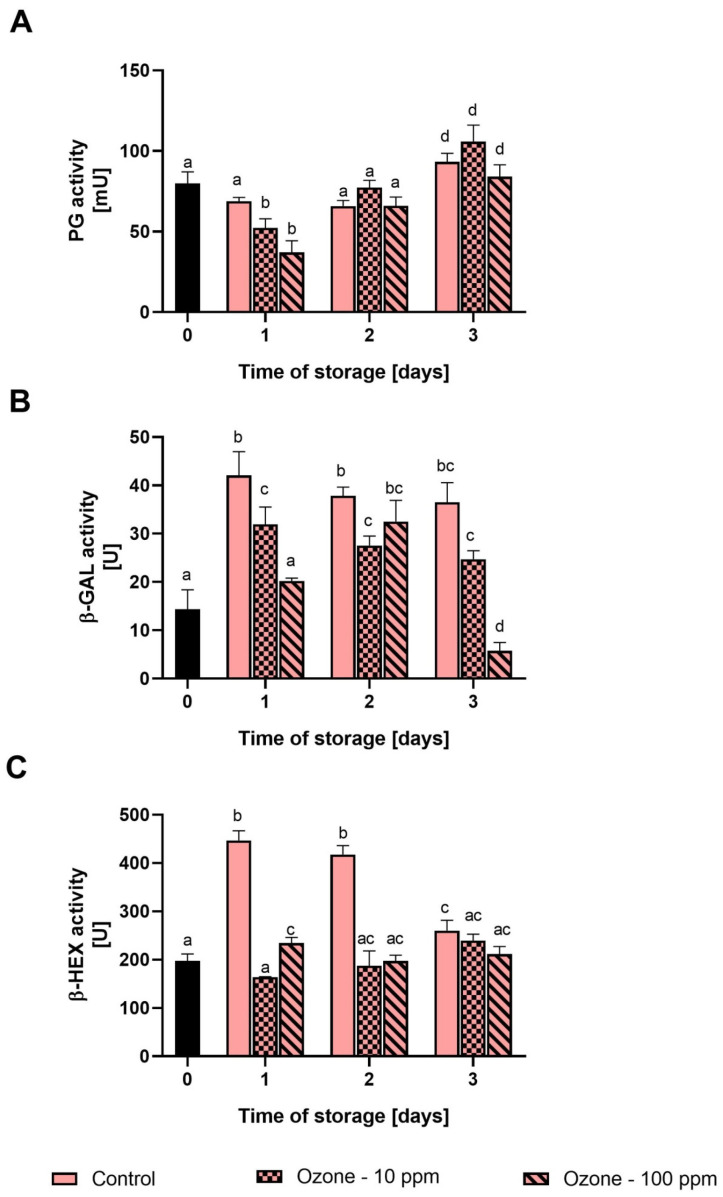
Effect of ozonation process on the activity of polygalacturonase (**A**), β-galactosidase (**B**) and β-hexosaminidase (**C**) in strawberry fruit during storage at room temperature. Mean values with standard deviations (error bars), with the same lower case are not statistically significant according to the t-Tukey test (α = 0.05).

The activity of enzymes directly related to texture changes in the fruit during storage is shown in Figure 2. In the course of the research, we found that the ozonation process as well as the storage time clearly influenced the activities of all enzymes analysed.

The activity of polygalacturonase after 1 day of storage decreased by 34% in fruit ozonated with gas at 10 ppm, and by 53% in fruit ozonated with gas at 100 ppm. In the control sample, we did not find any significant changes in the activity of this enzyme after 1 day of storage. After the second day of the experiment, the activity of PG in the control sample had not changed, while in the ozonated fruit it had increased sharply. After 3 days of storage, we found an increase in PG activity in all fruit samples.

The activity of β-galactosidase in the control sample after 1 day of storage increased sharply by 193% compared to the initial value. After days 2 and 3, the activity of β-Gal in the non-ozonated fruit had not changed significantly. In fruit ozonated with a gas concentration of 10 ppm, we found an increase in β-Gal activity of 122%, and in fruit treated with 100 ppm, no significant changes were found after 1 d of storage. After 2 and 3 days, the activity of β-Gal in fruit exposed to ozone at a concentration of 10 ppm had not changed significantly, while in fruit ozonated with 100 ppm, it had slightly increased, and on the last day it decreased sharply (to 6 U).

The activity of β-hexosaminidase increased sharply after 1 d of storage (by 127%) in non-ozonated fruit and remained at this level for another day. After 3 days, the β-Hex activity had decreased significantly to 260 U. In the case of ozonated fruit, a significant change was noted only in fruit ozonated with 100 ppm gas, after 1 day of storage (an increase of 19%). On the remaining dates of the experiment, the activity of β-Hex did not change significantly in ozonated fruit.

### 3.3. The Effect of Ozone Treatment on the Mitochondria Energy Metabolism in Strawberry Fruit

**Figure 3:**
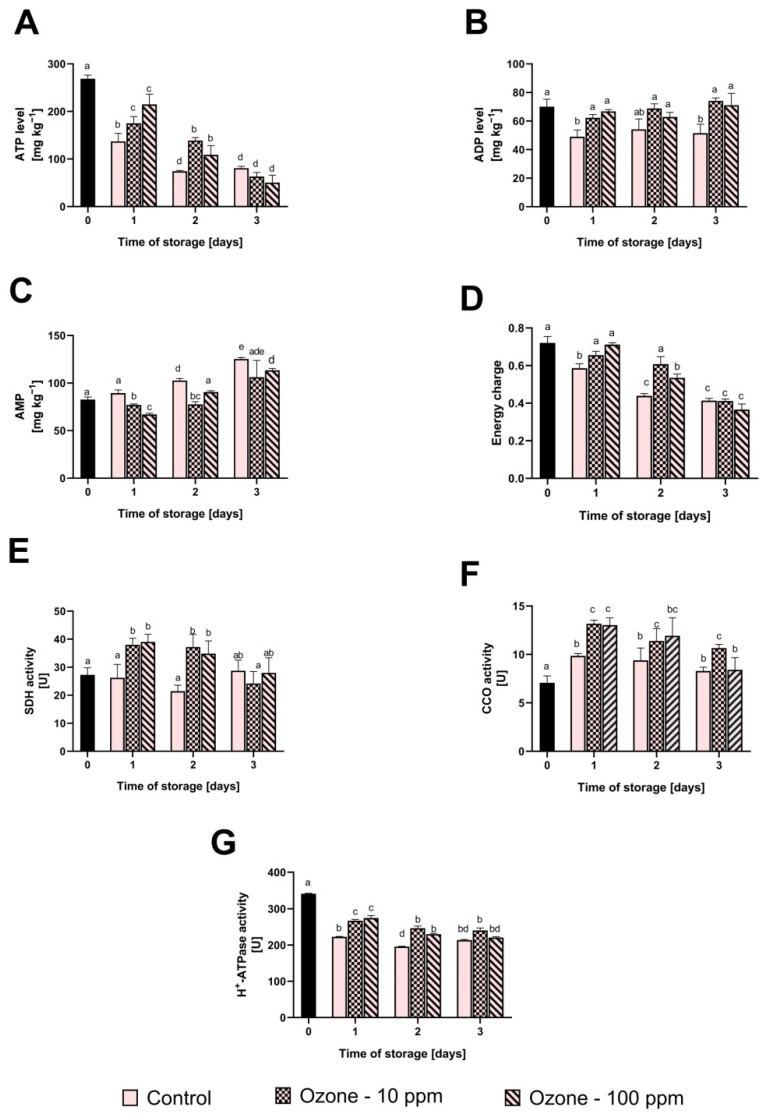
Influence of ozone treatment on the level of ATP (**A**), ADP (**B**), AMP (**C**), energy charge (**D**) and the activity of succinate dehydrogenase (**E**), cytochrome C oxidase (**F**) and H+ATPase (**G**) in strawberry fruit during storage at room temperature. Mean values with standard deviations (error bars), with the same lower case are not statistically significant according to the t-Tukey test (α = 0.05).

The levels of ATP, ADP, AMP, the energy charge of the fruit and the activity of enzymes involved in oxidative phosphorylation are shown in Figure 3.

The ATP content and the energy charge of the mitochondria in the control fruit gradually decreased with the extension of the storage period. On the last day of storage, we noticed a decrease in the ATP content of 73%, and in the energy charge of 42% compared to the initial value. The content of ADP in non-ozonated fruit decreased after 1 day of storage and had not changed at subsequent analysis dates, while the level of AMP showed a gradual increase with the extension of the storage time. In the case of ozonated fruit, with gas concentrations of both 10 and 100 ppm, we found a significantly lower rate of ATP decrease, which corresponded to the higher value of the energy charge of ozonated fruit after 1 and 2 days of storage. The ADP content in ozonated fruit did not change significantly during storage. On the other hand, the content of AMP in ozonated fruit was significantly lower than in the control fruit on all the dates of the analyses.

The activity of succinate dehydrogenase in the control fruit did not change significantly during storage, while in the ozonated fruit the activity of this enzyme increased sharply after 1 day and remained constant for the following day of the experiment. The activity of cytochrome oxidase in the control fruit slightly increased after 1 day of storage (39%) and remained constant over the following days of storage. In ozonated fruit, we noticed an initial, clear increase in CCO activity of 86 and 94% for 10 and 100 ppm, respectively, and a gradual decrease after 3 days of storage.

The activity of H+ATPase in the control fruit showed a noticeable decreasing tendency with the passage of the storage period. On the other hand, in the ozonated fruit, the rate of decline in the activity of this enzyme was clearly much lower. Both in the case of H+ATPase, as well as CCO and SDH, we found no significant influence of ozone concentration on the activity of this enzyme.

### 3.4. Changes in Mitochondrial Oxidative Stress Markers in Strawberry Fruit during Storage

**Figure 4:**
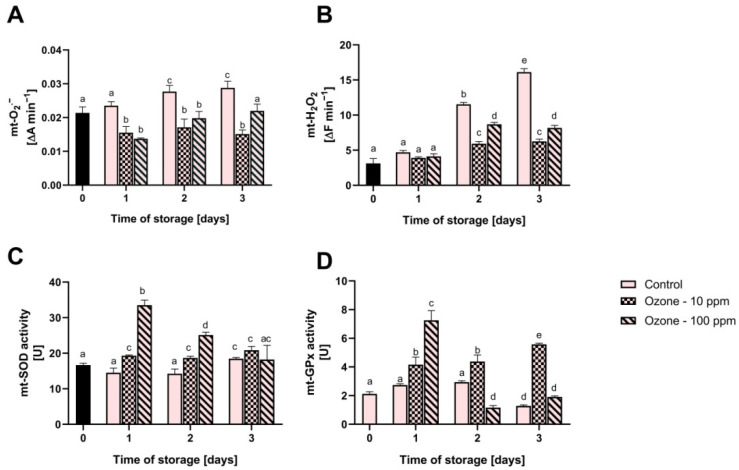
Effect of ozone treatment on the generation of mitochondrial superoxide radical anion (**A**) and hydrogen peroxide (**B**) as well as the activity of mitochondrial superoxide dismutase (**C**) and glutathione peroxidase (**D**) in fruit during storage. Mean values with standard deviations (error bars), with the same lower case are not statistically significant according to the t-Tukey test (α = 0.05).

The capacity of fruit mitochondria to produce reactive oxygen species (ROS) and the activity of selected mitochondrial antioxidant enzymes are shown in Figure 4.

In the course of the conducted research, we noticed a clear increase in the capacity of mitochondria to produce superoxide anion radical and hydrogen peroxide in non-ozonated fruit along with the extension of the storage period. On the final day of storage, the increase in the level of superoxide radical anion was 35%, and hydrogen peroxide 415%. Surprisingly, in the ozonated fruit, we found a lower mitochondrial ROS production capacity than in the control fruit. However, after 2 and 3 days of storage, the ROS level was higher in the fruit ozonated with 100 ppm gas than with 10 ppm gas.

The activity of superoxide dismutase in the fruit constituting the control sample did not change until the second day of storage. After this time, it slightly increased. In the case of fruit ozonated with gas at a concentration of 10 ppm, the SOD activity increased by 16% compared to the initial value and did not change significantly over the following days. On the other hand, the activity of mt-SOD in fruit ozonated with 100 ppm increased sharply after 1 day of storage by 101% and then it decreased slightly. Glutathione peroxidase showed an analogous tendency in ozonated fruit. In the control fruit, GPx activity did not change until the second day of storage, and after that time it significantly decreased.

### 3.5. The Level of MDA and Electrolyte Leakage in Ozonated Strawberry Fruit

**Figure 5:**
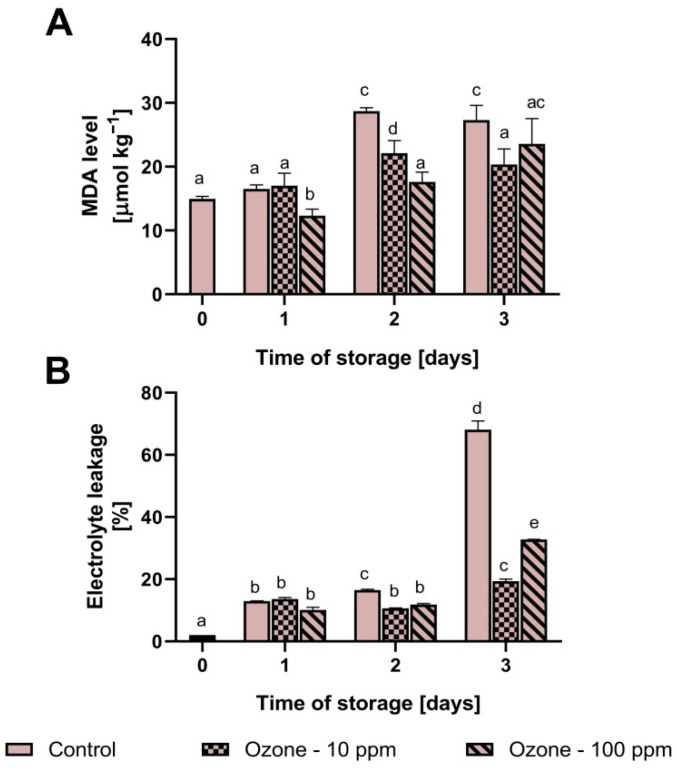
Effect of ozonation process on the level of malondialdehyde (**A**) and electrolyte leakage (**B**) in fruit during storage. Mean values with standard deviations (error bars), with the same lower case are not statistically significant according to the t-Tukey test (α = 0.05).

The content of malondialdehyde in the control sample increased with the extension of the storage period. After 3 days of storage, we noted an increase in lipid peroxidation products of 83%. In the case of the fruit ozonated with 10 ppm, we found no changes in the MDA content until after 1 day. After this time, the MDA concentration increased slightly and fell again to a value comparable to the initial value. In the sample ozonated at 100 ppm, we found a significant decrease in MDA after 1 day of storage and, over the remaining storage periods, a slight increase (Figure 5A).

The level of ion leakage from the fruit tissue is shown in Figure 5B. In the case of the control sample, we noticed a close relationship between storage time and ion leakage. After 3 days of storage, the ion leakage increased by 3255% compared to the initial value. We noticed a clear effect of ozone only after the second day of storage. At that time, ozonated fruit was characterised by lower ion leakage than the control fruit. For example, after the third day of storage, the leakage of ions in fruit ozonated with 10 ppm gas was lower by 71.5% compared to the non-ozonised sample, and in the case of 100 ppm it was lower by 51.8%.

## 4. Discussion

The firmness of berries, apart from the organoleptic characteristics and biological value, is one of the main attributes determining the suitability of the fruit for consumption, and it can also be an initial marker illustrating microbiological and biochemical changes occurring in the fruit during storage [36]. Fruit that is too soft is characterised by high susceptibility to mechanical damage, which contributes to an increased multiplication of microorganisms that cause fruit spoilage and rotting, thus significantly shortening the period of safe storage. Moreover, fruit that is too soft is negatively perceived by consumers and discourages purchase and consumption [26,37].

The texture of fruit is derived from their turgor pressure, the composition of the individual plant cell walls, and the middle lamina that holds the individual cells together [38]. The skin of the strawberry fruit is responsible for a significant part of the firmness of the fruit, which determines the values of the strength parameters, while the role of the subcutaneous and parenchymal tissue is small, but not insignificant [3]. For most plant materials, the skin (epidermal cells plus epidermis) is a stiffer structure than the internal tissues. On the contrary, in the case of strawberry fruit, which is characterised by a weaker skin, the mechanical role of the sub-epidermal cells is also important. Strawberry fruit is not an isotropic material, and its structure varies depending on the type of tissue (epidermis, subcutaneous tissue, cortical cells, vascular bundles and core). The force-displacement curve at small deformations is determined not only by the mechanical properties of the skin, but also of other tissues. At the cellular and tissue level, there are three main structural factors that contribute to the mechanical properties of plant materials. They are: the turgor pressure in individual cells (i.e., the force exerted on the cell membrane by the intracellular fluid), the stiffness of the cell wall, and the cell–cell adhesion, determined by the integrity of the middle lamina and plasmodesmata [36]. Our research has shown that the process of cyclic ozonation of strawberry fruit with gas at a concentration of both 10 and 100 ppm contributes to the improvement of texture parameters during storage at room temperature. Moreover, it turned out that fruit ozonated with a lower concentration gas had better mechanical parameters. This is consistent with many other scientific reports. For example, in the work of Chen et al. [39], the effect of weekly ozonation of strawberries stored at 4 °C for 10 h with gas at concentrations of 2.144, 6.432 and 10.72 mg m^−3^ on their firmness and biochemical parameters was determined. The authors observed that all ozone concentrations applied reduced the loss of firmness during storage, and clearly noticed that the fruit ozonated with the lowest concentration (2.144 mg m^−3^) had the best firmness. A similar effect was noted by Ali et al. [40] who ozonated papaya fruit with gas at concentrations of 1.5, 2.5, 3.5 for 96 h prior to 14-day storage at room temperature. However, they found that the fruit ozonated with 5 ppm gas showed significantly worse firmness than the control sample. There are also few studies, showing that the single and short-term ozone treatment correlates with the maintenance of the good texture of strawberries during storage. Panou et al. [41] proved that the fruit treated with ozone at a concentration of 1 ppm for 40 min were characterised by much better firmness than the control sample, as well as fruit ozonated under other process conditions (0.5 and 1.5 ppm). Aday et al. [7] have found a significant lower decrease in firmness, springiness, cohesiveness and chewiness in strawberries immersed in water saturated with ozone (0.075 mg L^−1^) than in the control during 4-week storage. Moreover, they have noticed that the application of ultrasound (power 30 W) and chlorine dioxide (6 mg L^−1^) enhances the positive effect of ozonated water. Proper turgor leads to stiffness of cells and tissues, and together with the cell wall provides mechanical support to maintain the shape of the fruit.

Post-harvest softening is one symptom of the progressive aging of fruit. When this process occurs, a major role is played by a number of biochemical and molecular changes related to the damage of cell membranes along with the loss of cell turgor, decomposition and depolymerisation of the components constituting the building blocks of the cell wall, i.e., pectin, hemicellulose and cellulose, as well as reduction in the contact between cells and plasmolysis [42]. Additionally, Zhou et al. [29,34] indicate that one of the reasons for the deterioration of fruit firmness is the loss of antioxidant properties and the occurrence of oxidative stress in the plant cell.

The immediate cause of the deterioration of fruit firmness after harvesting is the increased activity of enzymes hydrolysing the components of the cell wall, e.g., polygalacturonase, β-galactosidase and β-hexosaminidase, both endo- and exogenous, produced by microorganisms inhabiting the surface of the fruit. Polygalacturonase is responsible for the hydrolysis of α-1,4-glycosidic bonds between galacturonic acid residues in pectins [43]. The role of β-galactosidase is, in turn, to remove non-reducing β-D-galactosyl residues in hemicelluloses and pectins, which increases the porosity of the cell wall and facilitates the access of other enzymes that degrade the cell wall [42]. β-hexosaminidase cleaves terminal *N*-acetyl-D-hexosamine residues from *N*-glycoproteins, which are also present in the plant cell wall [27]. In the course of further research, we found that ozonated fruit is characterised by a reduced activity of PG (only after 1 day of storage) as well as β-Gal and β-Hex on all analysis dates. In the work by Chen et al. [39] cited above it was shown that ozonation decreased the expression of genes encoding enzymes involved in the breakdown of cell wall components, i.e., pectinase, cellulase, endoglucanase and pectinesterase. Therefore, we can assume that the lower catalytic capacity of PG, β-Gal and β-Hex directly contributed to better hardness of ozonated fruit than non-ozonated fruit.

Recently published research shows that the preservation of high-quality plant materials during storage is related to their energy metabolism [44]. Adenosine triphosphate (ATP) produced by mitochondria through oxidative phosphorylation is a source of the energy needed to carry out many biochemical processes necessary for the cell to function properly and to adapt it to various conditions of both abiotic and biotic stress [44,45]. It turns out that ensuring a high level of ATP in the cell through the use of various elicitors for the post-harvest processing of fruit and vegetables contributes to the maintenance of the proper integrity of membranes, increases the activity of antioxidant enzymes and stimulates the activity of the phenylpropanoid pathway, leading to an increase in the antioxidant status of the cell [44,46,47]. Moreover, the increased adenylated energy charge corresponds to the decreased expression of genes that degrade the cell walls, stimulated by the biosynthesis of phytoalexins, PRs proteins and plant hormones [44,46]. As a consequence, horticultural crops are characterised by an extended period of storage, while maintaining their high quality [44].

The following enzymes play an important role in maintaining the correct energy metabolism of mitochondria: succinate dehydrogenase (SDH), cytochrome C oxidase and H+ATPase. SDH is an enzyme of respiratory chain complex II that catalyses the succinate dehydrogenation reaction, leading to the formation of fumarate [48]. Cytochrome C oxidase (respiratory chain complex IV) collects electrons from cytochrome c and transfers them to an oxygen molecule, reducing it, resulting in the formation of two water molecules when H+ ions are attached [49]. In turn, H+ATPase accelerates the breakdown of ATP into ADP, releasing the energy needed to establish a transmembrane electrochemical gradient and transport protons across the membrane during ATP biosynthesis [50]. Moreover, many studies undertaken so far have shown that ATP and ADP content and the value of the energy charge is closely correlated with the activity of the above enzymes [44]. We have also noticed this regularity in the presented research. Fruit ozonated with gas at a concentration of 10 ppm was characterised by a higher activity of SDH, CCO and H+ATPase throughout the storage period, which corresponded to a higher level of ATP and energy charge. On the other hand, increasing the gas concentration to 100 ppm produced the same effect only up to the second day of storage. After this time, the values of the analysed indices did not differ from the non-ozonated sample. An improvement of the energy metabolism of ozonated fruit was also found in the work of Piechowiak et al. [45] in raspberry fruit cyclically ozonated with gas at a concentration of 8–10 ppm (30 min) every 12 h for 3 days of storage at room temperature, as well as in the work by Piechowiak [48], in which an increase in ATP levels was found in raspberry, strawberry, blackcurrant, blackberry and highbush blueberry fruit after a single ozonation process (15 ppm, 30 min). Aghdam et al. [44] in their work explain the role of the energy metabolism of fruit treated with various elicitors in shaping their post-harvest quality. The authors indicate that an increased capacity of mitochondria to biosynthesise ATP may result directly from the activation of genes encoding mitochondrial enzymes. Moreover, increased ATP biosynthesis may be mediated indirectly by the activation of NAD kinase, catalysing the phosphorylation of NAD+ to NADP+, which is then converted to NADPH, used in the respiratory chain, by increased activity of the pentose-phosphate pathway and/or EMP. Sachadyn-Król and Agriopoulou [9] describe the capacity of ozone to penetrate into the plant through the stomata into the aqueous apoplast, where it immediately breaks down into reactive oxygen species that mediate many signal transduction pathways, leading to the activation of genes involved in protecting the plant against environmental stress. Therefore, it should be assumed that ozone and/or its decomposition products could activate the above mechanisms. However, to fully explain them, further research is needed, including at the molecular level.

Apart from peroxisomes, mitochondria are the main place of production of reactive oxygen species in the plant cell [51]. The superoxide anion radical is formed during the electron reduction of oxygen in respiratory chain complexes I, II and III, which can then be reduced to hydrogen peroxide by the action of superoxide dismutase [52]. Peroxidases present in the plant cell and low molecular weight antioxidants can ultimately neutralise the hydrogen peroxide produced [52]. According to Zhou et al. [29,34], maintenance of an appropriate redox state in the cell is one of the key determinants of fruit firmness during storage. The loss of the antioxidant properties of fruit is associated with an increased generation of reactive oxygen species that have the capacity to oxidise unsaturated fatty acids, which are a component of cell membranes, leading to loss of membrane integrity, changes in permeability and, as a result, loss of turgor [53]. Malondialdehyde levels and the degree of leakage of electrolytes from the tissue are often used as markers for cell membrane integrity [44].

In our research, we showed that the mitochondria of ozonated strawberries produced less ROS than non-ozonated fruit. This was probably due to the stimulation of the biosynthesis of mitochondrial antioxidant enzymes as a result of ozone elicitation of the fruit, and ultimately, the higher catalytic activity of the SOD and GPx proteins. Ultimately, the higher antioxidant capacity of mitochondria translated into lower levels of MDA and the degree of ion leakage in the ozonated fruit. However, as can be seen in Figure 4, the level of MDA and ion leakage in the fruit ozonated with 100 ppm gas was higher after the third day of storage than in the fruit ozonated with 10 ppm, which could be due to the cytotoxic effect of ozone and damage to the defence mechanisms observed as a sharp decrease in SOD and GPx activity after 2 days of storage, as well as worse mechanical parameters. It should, therefore, be assumed that the beneficial effect of ozonation with regard to the texture of the fruit is obtained by using a gas with a concentration of 10 ppm.

## 5. Conclusions

In conclusion, the ozonation process significantly prevented the loss of fruit texture during storage at room temperature. The positive effect of ozone was directly related to the inhibition of the activity of enzymes involved in the degradation of the fruit cell walls, i.e., polygalacturonase, β-galactosidase and β-hexosaminidase, as well as indirectly from the improved energy metabolism of the fruit. The higher content of ATP and adenylated energy charge corresponded to the higher resistance of ozonated fruit to abiotic stress, leading to the maintenance of the integrity of cell membranes and, consequently, to maintaining good hardness of the fruit throughout the storage period. However, in order to fully understand these mechanisms, it is necessary to conduct deeper analysis, especially at a molecular level.

## Data Availability

Data is contained within the article.
